# Interfacial Doping Effects in Fluoropolymer-Tungsten Diselenide Composites Providing High-Performance P-Type Transistors

**DOI:** 10.3390/polym13071087

**Published:** 2021-03-30

**Authors:** Hyeonji Lee, Seongin Hong, Hocheon Yoo

**Affiliations:** 1 Department of Electronic Engineering, Gachon University, 1342 Seongnam-daero, Seongnam 13120, Korea; bcd10@gachon.ac.kr; 2 School of Advanced Materials Science and Engineering, Sungkyunkwan University, Sunwon 16419, Korea

**Keywords:** WSe_2_, Cytop, transition metal dichalcogenides, annealing process, p-type transistor

## Abstract

In this study, we investigated the p-doping effects of a fluoropolymer, Cytop, on tungsten diselenides (WSe_2_). The hole current of the Cytop–WSe_2_ field-effect transistor (FET) was boosted by the C–F bonds of Cytop having a strong dipole moment, enabling increased hole accumulation. Analysis of the observed p-doping effects using atomic force microscopy (AFM) and Raman spectroscopy shed light on the doping mechanism. Moreover, Cytop reduces the electrical instability by preventing the adsorption of ambient molecules on the WSe_2_ surface. Annealing Cytop deposited on WSe_2_ eliminated the possible impurities associated with adsorbates (i.e., moisture and oxygen) that act as traps on the surface of WSe_2_. After thermal annealing, the Cytop–WSe_2_ FET afforded higher p-type conductivity and reduced hysteresis. The combination of the Cytop–WSe_2_ FET with annealing provides a promising method for obtaining high-performance WSe_2_ p-type transistors.

## 1. Introduction

Transition-metal dichalcogenides (TMDs) are used as channel materials that can overcome the limitations of existing silicon devices, with controllable bandgaps, atomically thin 2D structures, and compact metal and chalcogen lattice structures [1,2,3]. The ability to control the channel thickness at the atomic level can improve gate control over the channel barrier and reduce short-channel effects, an issue inherent in silicon. Tungsten diselenide (WSe_2_) is a TMD material, the ambipolar transport characteristics [4] of which can be adjusted by choosing a suitable contact metal [5,6,7] and the number of layers [8,9], thereby affording excellent optical properties with high quantum efficiency [10].

However, the WSe_2_ transistor itself has intrinsic internal defects due to Fermi level pinning with the contact metal electrode, and impurities caused by the device fabrication processes. Furthermore, because the Fermi level of WSe_2_ is close to the middle of the bandgap [11], it is difficult to inject holes or electron carriers between the contact metal and WSe_2_. These issues limit the effective carrier mobility and lead to poor process yields and non-uniform properties [12]. To address these issues, an appropriate doping method must be used to control the electrical properties of WSe_2_.

However, an appropriate method for doping TMDs, including WSe_2_, is still lacking. Conventional doping techniques such as ion implantation are not compatible with TMDs, as these processes cause significant damage to the crystal structure of TMDs. One approach for fabricating doped TMDs is by replacing the transition metals or chalcogen atoms with other atoms. Another approach involves doping with gas. When TMDs are exposed to NO_2_ or K gas, their electrical properties can be adjusted depending on the exposure concentration or time [13,14]. However, these methods involve complex processes and are limited in that the doping effect cannot be stably maintained for a sufficiently long time.

In this study, we present a simple p-doping technique in which Cytop is spin-coated on top of WSe_2_, forming a fluoropolymer–WSe_2_ composite (F-WSe_2_ devices), in which the electrical polarity of multilayer WSe_2_ can be successfully controlled. By annealing at temperatures (*T_A_*) of 100, 200, and 300 °C, the carrier mobility is enhanced 20-fold, affording a maximum mobility of 112 cm^2^ V^−1^s^−1^. Compared to the pristine WSe_2_ devices, the F-WSe_2_ device shows a significant improvement in the on current (≈6 × higher) and off current (≈9 × 10^−4^ × lower) at *T_A_* = 200 °C, where the device exhibits high-performance metrics: *μ_hole_* = 85 cm^2^ V^−1^s^−1^ and *I_on_*/*I_off_* = 1.08 × 10^6^. Raman spectroscopy and atomic force microscopy (AFM) are also employed to account for the observed p-doping effects in the F-WSe_2_ devices. The high annealing temperature causes molecular aggregation in Cytop, providing a strong p-type doping effect due to the higher density of the C–F dipole domains [15,16]. Furthermore, Cytop prevents the penetration of moisture and other contaminants into the F-WSe_2_ devices [17]. As a result, the observed p-doping effect is effectively maintained with negligible changes. The high-performance characteristics of the p-doped WSe_2_ transistor are maintained for 25 days under ambient atmosphere (only 4% variation in *V_th_* and 19% variation in *μ_hole_* due to air-exposure effects).

## 2. Materials and Methods

### 2.1. Device Fabrication and Measurements

Multilayer WSe_2_ flakes were mechanically exfoliated from bulk WSe_2_ (SPI crystals) using Scotch tape. The exfoliated WSe_2_ flakes were transferred onto a Si substrate with 300 nm-thick thermal SiO_2_. To remove the chemical residue that remained after the transfer process, the Si substrates were immersed in acetone, rinsed with isopropyl alcohol, and dried. On a SiO_2_/Si substrate with the WSe_2_ flakes transferred, the source and drain electrodes were patterned using photolithography and the lift-off method. An e-beam evaporator was used to deposit 20 nm titanium and 100 nm gold. The p-doped Si was used as a back gate by applying silver paste. The fabricated WSe_2_ transistor was doped with the fluorinated polymer Cytop (solution:solvent = 1:10) for 60 s at 3000 rpm by a general spin-coating process. The annealing process was then performed at 100, 200, and 300 °C for 30 min. The thickness of the spin-coated Cytop was approximately 14 nm.

### 2.2. Film Analysis

Surface images and line profiles of the Cytop-doped WSe_2_ transistors annealed at different temperatures (i.e., *T_A_* = 100, 200, and 300 °C) were acquired using AFM (XE7 Atomic Force Microscope, Park Systems, Korea) in non-contact mode. The dependence of the Raman spectra of the WSe_2_ film on the annealing temperature was analyzed using an ALPHA300 (WITec Co., Germany) instrument with laser excitation at 532 nm, which has a resolution of about 1.1 cm^−1^ at 1800 lines mm^−1^ grating. The 532 nm laser line is often used to investigate the doping behaviors of TMDs due to the resonance Raman scattering by this wavelength [18,19,20,21]. The power of the excitation was 1 mW to minimize the heating effect [18].

### 2.3. Air-Stability Characterization

The F-WSe_2_ device was doped with Cytop and annealed at 200 °C in a clean room with a relative humidity (RH) of 25% at room temperature (25 °C). Transfer curves were acquired every five days up to the 25th day. All electrical characteristics were measured in air.

### 2.4. Extraction of Parameters to Evaluate Electrical Performance

Hole mobility (*μ_hole_*) is one of the parameters for evaluating the performance of transistors. The mobility of pristine WSe_2_ and F-WSe_2_ (*T_A_* = 100, 200, and 300 °C) is extracted from the drain current plots of the transfer curves at *V_ds_* = −1 V, using the Equation (1) for the linear region.

(1) $$\mu_{hole}=\frac{\partial \left|I_{\mathrm{ds}}\right|}{\partial \left|V_{gs}\right|}\cdot \frac{L}{w}\frac{1}{C_{\mathrm{ox}}|V_{ds}|\;}$$

where C_ox_ is the back-gate capacitance of the SiO_2_, and L and W are the WSe_2_ channel length and width, respectively.

The on/off current ratio is calculated as *I_on_*/*I_off_*, which is the ratio of *I_off_*, which is the drain current in the off-state region under the threshold voltage (*V_th_*) value, and *I_on_*, which is the drain current in the on state at *V_gs_* = −40 V.

## 3. Results and Discussion

### 3.1. F-WSe_2_ Electronic Devices

**Figure 1:**
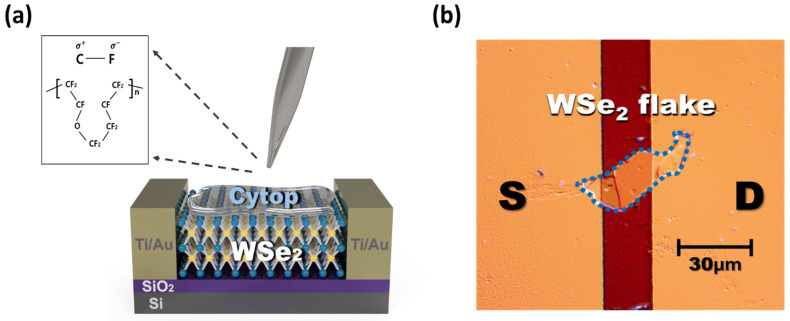
(**a**) Schematic of the fluoropolymer–WSe_2_ composite (F-WSe_2_) device. Inset: Chemical structure of Cytop; (**b**) optical microscopy (OM) image of the F-WSe_2_ device flake.

Figure 1a indicates a schematic of the F-WSe_2_ device. Cytop was spin-coated on the WSe_2_ device in the pristine state and annealed at various annealing temperatures (*T_A_* = 100, 200, and 300 °C). The inset of Figure 1a shows the chemical structure of Cytop. The C–F bond possesses a dipole moment, which accumulated holes and depleted electrons, enabling p-doping enhancement in the WSe_2_ channel interface [22].

Figure 1b presents an optical microscope (OM) image showing the top-view of the F-WSe_2_ device. Owing to the two-dimensional bonded structure of WSe_2_ inorganic materials, these materials are greatly affected by the Cytop doping effect without any effects of variations such as grain boundary traps. As shown in the OM image, the doping process was performed by spin-coating Cytop without damaging or physically affecting the crystallinity of WSe_2_. WSe_2_ flakes were used as the channel for the transistor, and a device was manufactured with a channel length of 20 μm, a width of 17.37 μm, and a thickness of 43.61 nm (Figure S1).

### 3.2. Electrical Characteristics

For quantitative comparison, the same device was characterized before and after the Cytop doping process to investigate the dependence of the change in the WSe_2_ electrical properties. After plotting the transfer curves (I_ds_–V_gs_) and output curves (I_ds_–V_ds_) of the pristine WSe_2_ device described previously, F-WSe_2_ devices annealed at *T_A_* = 100, 200, and 300 °C for 30 min were sequentially analyzed.

**Figure 2:**
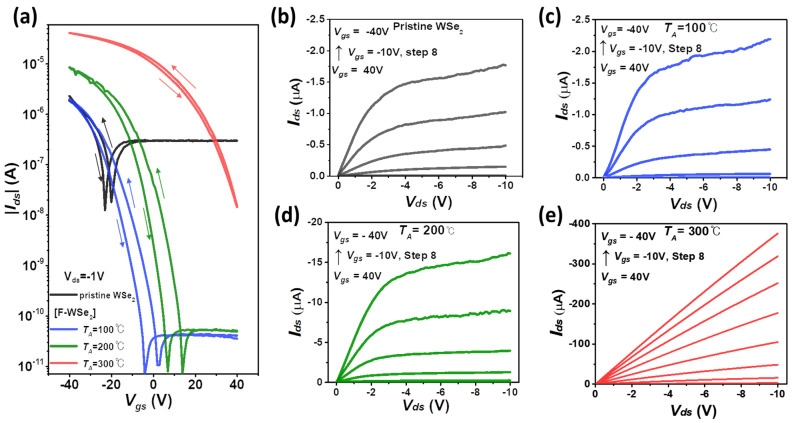
Electrical characteristics of the tungsten diselenide (WSe_2_) device. (**a**) Change in transmission curve for *T_A_* = 100, 200, and 300 °C for the pristine WSe_2_ device and F-WSe_2_ device; (**b**) output curve of pristine WSe_2_ transistor; output curve of F-WSe_2_ transistor annealed at *T_A_* = (**c**) 100 °C, (**d**) 200 °C, and (**e**) 300 °C.

Figure 2a presents a comparison of the transfer curves (I_ds_–V_gs_) of the pristine and F-WSe_2_ devices. The pristine device exhibits the typical transfer characteristics of ambipolar charge transport (V-shaped curve). The on current of the pristine WSe_2_ device before doping was 2.30 × 10^−6^ A, and the off current was 1.26 × 10^−8^ A. When *T_A_* = 100 °C, the on current was similar to that obtained with the pristine sample, but the off current declined significantly to 8.46 × 10^−12^ A. For the sample annealed at *T_A_* = 200 °C, the on current was 8.52 × 10^−6^ A. It is thought that the p-type current increased significantly at negative bias due to hole carrier transmission through Cytop, and the n-type current and electron carrier transmission decreased at positive bias. In addition, when the *T_A_* was increased after doping, the threshold voltage (V_th_) gradually shifted to the positive direction, indicating that the Cytop molecules acted strongly as p-type dopants [23,24]. This phenomenon may be related to the electric dipole moment of the C–F bond at the end groups of the Cytop-encapsulating molecule [25,26,27]. At *T_A_* = 300 °C, the on current was 4.10 × 10^−5^ A. Compared to the pristine WSe_2_ device, the on current (≈6× higher) and off current (≈9 × 10^−4^ × lower) were significantly improved for the device annealed at *T_A_* = 200 °C. The high-temperature annealing (*T_A_* = 300 °C) enabled the pristine WSe_2_ film to be highly doped and caused metallic-like behavior, which is a result consistent with the previous report [23]. The metallic-like behavior of the pristine WSe_2_ device at *T_A_* = 300 °C still suffered from a significantly low on/off current ratio (~70), high off-current (3.15 × 10^−6^ A), and hysteresis (Figure S2). In the output curve at *T_A_* = 300 ℃, we observed metallic-like behavior. Owing to the highly shifted V_th_, the only linear region appeared in the operating voltage −40 V < V_g_ < 40 V, exhibiting no saturation characteristics.

The output characteristics (I_ds_–V_ds_) of the pristine WSe_2_ device and the F-WSe_2_ device according to the annealing temperature (*T_A_*) are shown in Figure 2b–e. For the samples annealed at *T_A_* = 100 and 200 °C after doping with Cytop, the devices showed clear unipolar p-type behavior (Figure 2c,d). In addition, compared to pristine WSe_2_, the negative I_ds_ of F-WSe_2_ became more negative as the *T_A_* increased. This is because a dipole moment is induced on Cytop, and the channel conductance increases as the width of the Schottky barrier decreases. Based on the above investigations, we concluded that the optimal annealing temperature was *T_A_* = 200 °C, which exhibited the optimized on/off current ratio and carrier mobility as high as *μ_hole_* = 85 cm^2^ V^−1^s^−1^ and I_on_/I_off_ = 1.08 × 10^6^.

**Figure 3:**
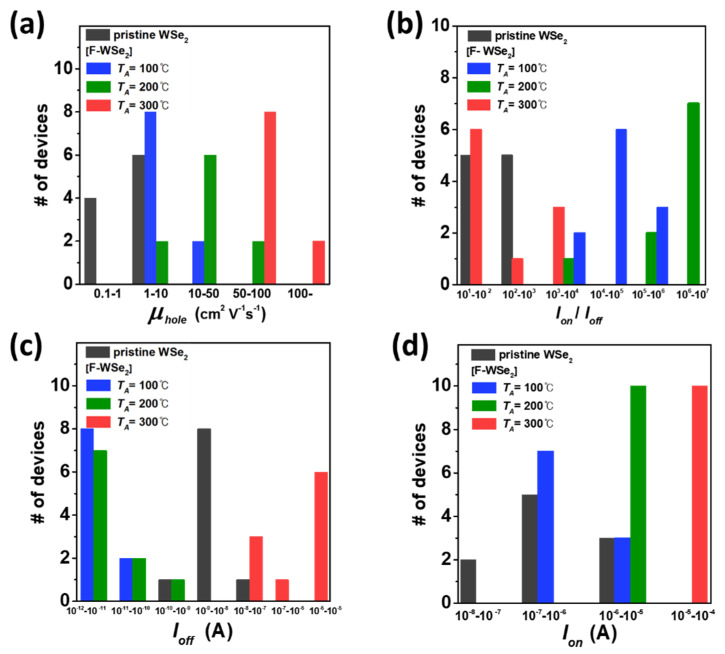
Histogram exhibiting statistical distributions of (**a**) carrier mobility, (**b**) on/off current ratio, (**c**) off current, (**d**), on current.

To statistically evaluate the variation in the effect of Cytop doping on the WSe_2_ device, ten pristine WSe_2_ devices were characterized, and the F-WSe_2_ devices were annealed at *T_A_* = 100, 200, and 300 °C, respectively, after Cytop doping. Figure 3a clearly shows that the higher the *T_A_*, the stronger the Cytop doping effect, resulting in a higher *μ_hole_*.

Figure 3b–d shows a histogram of the device number against the current level (on–off current, on/off ratio). The histograms were divided into increments of 10. In the I_on_/I_off_ histogram (Figure 3b), I_on_/I_off_ increased significantly at *T_A_* = 100 and 200 °C after doping. However, at *T_A_* = 300 °C, the Cytop doping effect was strong, leading to rapid p-doping, and the on/off ratio (I_on_/I_off_) was lower than those at *T_A_* = 100 and 200 °C. This is because the on and off currents both increased owing to excessive p-doping. This trend was confirmed by the histograms of I_on_ and I_off_ for each of the ten devices (Figure 3c,d). At *T_A_* = 100 and 200 °C, the devices exhibited an off current and improved on current compared to the pristine device. The transfer curves for the ten devices are shown in Figure S3.

### 3.3. Variation of Cytop Microstructure and Chemical Composition with Annealing Temperature

**Figure 4:**
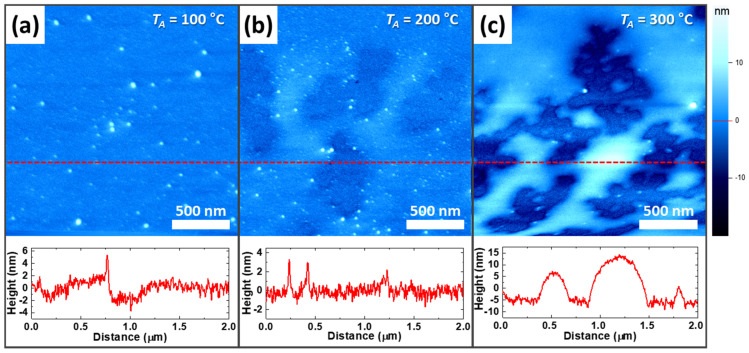
Atomic force microscopy (AFM) surface analysis of Cytop-coated Si substrate. AFM mappings and height graphs of Cytop-coated Si substrate annealed (**a**) at *T_A_* = 100 °C, (**b**) at *T_A_* = 200 °C, and (**c**) at *T_A_* = 300 °C.

Atomic force microscopy (AFM) and Raman spectroscopy measurements were performed to analyze the effect of Cytop doping. To further investigate the morphological properties of Cytop depending on the annealing temperature, the surface film properties were characterized using AFM, as shown in Figure 4. Thus, it can be seen that the particle size increased with the annealing temperature. The samples annealed at *T_A_* = 100 °C did not show well-defined domains (Figure 4a). At *T_A_* = 200 °C, the domains gradually became visible (Figure 4b). At *T_A_* = 300 °C, the domain size increased and the domains became clear (Figure 4c). In other words, with increasing annealing temperature, the surface changed clearly in terms of the size and shape of the domains [28]. These results indicate that the C–F bond of Cytop increases the overlap of the molecular dipole moment, as fewer impurities remain at higher temperatures.

**Figure 5:**
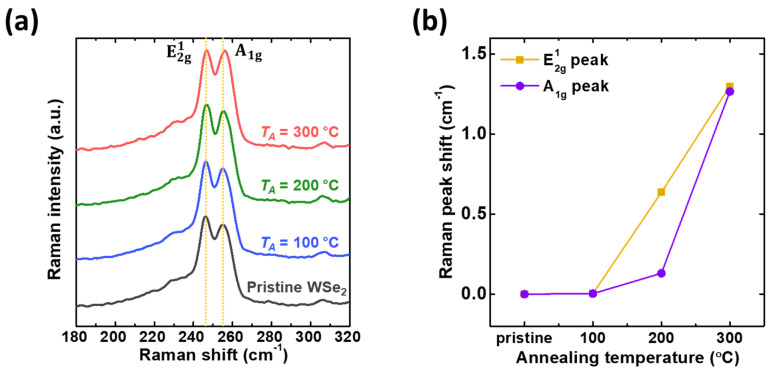
(**a**) Raman spectra of pristine WSe_2_ and F-WSe_2_ (*T_A_* = 100, 200, and 300 °C) and (**b**) Raman peak shift data for E_2g_^1^ and A_1g_ modes.

Raman spectroscopy is a non-destructive tool used to investigate doping effects in 2D materials [29]. As evidence of the p-doping effect by Cytop, the blueshift of the Raman peak after Cytop formation on the WSe_2_ appears to be related to the p-doping phenomenon, which is a result consistent with previous p-doping effects on TMDs [22,30]. To be specific, n-doping leads to softening and a decrease in the strength of the A_1g_ phonon, while p-doping causes a blueshift and an increase in the intensity of the A_1g_ phonon [31,32,33,34]. Figure 5a shows the Raman spectra of F-WSe_2_ and pristine WSe_2_ as a function of the *T_A_*. The A_1g_ peak clearly gained intensity for the sample treated at *T_A_* = 300 °C. These results indicated that F-WSe_2_ was p-doped. For further investigation, the degree of shift of the peaks of the A_1g_ and E^1^_2g_ modes was investigated (Figure 5b). The peak of the A_1g_ mode appeared at 255.05 cm^−1^ for pristine WSe_2_ and at 256.31 cm^−1^ for F-WSe_2_ (*T_A_* = 300 °C), representing a blueshift of 1.26 cm^−1^. Similarly, the peak of the E^1^_2g_ mode was also blueshifted by 1.25 cm^−1^. Based on the above results, we conclude that the peak corresponding to the A_1g_ phonon shows a blueshift and increased intensity in the case of F-WSe_2_ compared to the pristine sample. This trend is more pronounced with increasing *T_A_*, indicating that higher levels of p-doping in WSe_2_ can be achieved at higher *T_A_*.

### 3.4. Doping Mechanism

**Figure 6:**
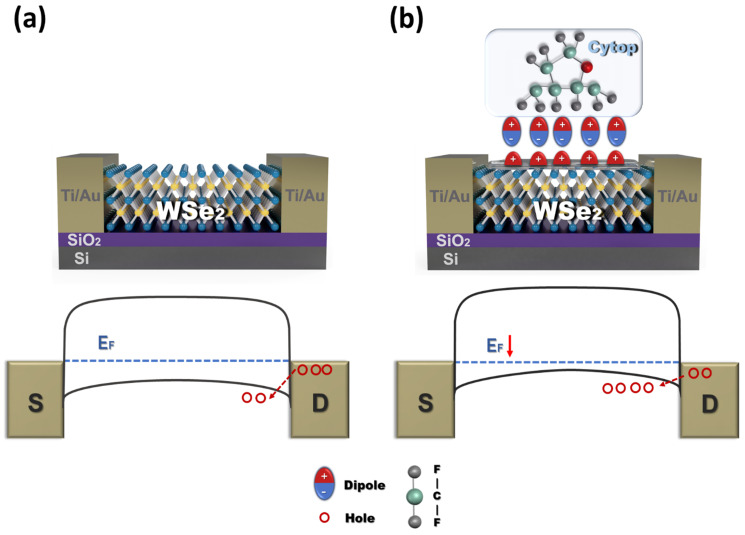
Schematics illustrating Cytop doping effects. Schematic and energy band diagram of the equilibrium state before and after doping with the Cytop layer. Cytop is a fluoropolymer with many C–F bonds. The dipole in Cytop induced by the C–F bond leads to holes in WSe_2_.

The Fermi level of undoped WSe_2_ is close to the middle of the bandgap; thus, the channel of the transistor forms a large hole barrier with the contact metal (Figure 6a). Therefore, the hole injection is restricted. On the other hand, when WSe_2_ is coated with Cytop, the C–F bond, which generates an electric dipole near the WSe_2_ interface, causes greater hole accumulation. The generated electrical dipole can lead to hole accumulation by creating a negative pole on the WSe_2_ channel surface (Figure 6b). As holes accumulate in the WSe_2_ channel, the Fermi level decreases [35]. As a result, the hole injection barrier with the contact metal is also reduced, and the hole mobility can increase at the same negative voltage bias as before doping.

The higher the thermal annealing temperature, the larger the movement of the polymer chains of Cytop, and the fluorine atoms can be rearranged on the WSe_2_ surface [22]. Thus, depending on the thermal annealing temperature, more dipole moments can be induced as the C–F bonds are more aligned on the WSe_2_ surface, resulting in a large downward shift of the Fermi level, and the p-doping effect thus becomes stronger. As *T_A_* increased, impurities (e.g., moisture) on the WSe_2_ surface decreased and the C–F bonds in Cytop became more densely aligned to the WSe_2_ surface, resulting in no hysteresis and highly p-doped characteristics. In conclusion, evaluation of the electrical characteristics and doping mechanism of transistors indicates that the degree of induction of the dipole on Cytop varies depending on the annealing temperature; thus, the charge transport of the channel and the degree of the p-doping effect can be controlled.

### 3.5. F-WSe_2_ Device Performance Based on Doping Effect

**Figure 7:**
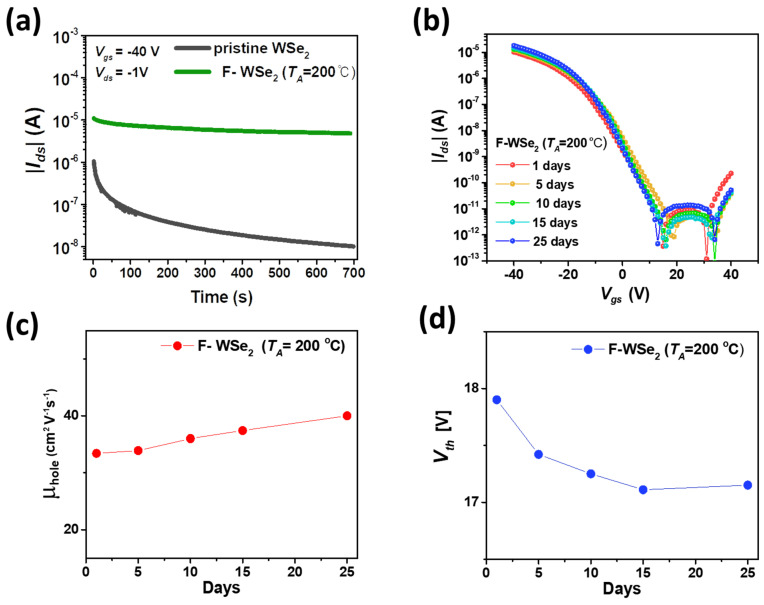
Stability test of F-WSe_2_. (**a**) Reduction in bias stress due to passivation effect due to Cytop doping. (**b**) F-WSe_2_ (*T_A_* = 200 °C) transfer characteristics of the device with increasing time stored in air at room temperature. All electrical characteristics were measured in air. (**c**) Threshold voltage and (**d**) hole mobility change of F-WSe_2_ device annealed at *T_A_* = 200 °C after Cytop doping for 25 d in air.

Figure 7a shows the negative bias stress (NBS) data for the pristine WSe_2_ device and F-WSe_2_ device. The F-WSe_2_ device was annealed for 30 min at *T_A_* = 200 °C after coating Cytop on the pristine WSe_2_ device. By performing the bias stress test, the decay of the electrical properties over time can be monitored to evaluate the trapping effect in the device. This effect was visualized by applying V_gs_ = −40 V and V_ds_ = −1 V for 700 s. The current level in the pristine sample decreased continuously, whereas the current level remained almost constant after Cytop doping. The large amount of fluorine contained in Cytop can diffuse into the WSe_2_ channel during annealing at 200 °C, and thus the stability of the transistor can be greatly improved through fluorine diffusion [36,37,38]. In addition, the Cytop polymer has low polarity and no OH group, which shows that it can efficiently remove trapping sites due to moisture [39,40]. As a result, the WSe_2_ transistor subjected to Cytop doping and subsequent annealing maintained a more stable state under the gate bias stress.

As shown in Figure 7b, the device was stored under ambient atmosphere to evaluate the effect of Cytop on the lifetime (or long-term stability) of the F-WSe_2_ device (*T_A_* = 200 °C). The electrical properties of the device were measured for 25 d in air. The on current increased slightly over time. The observed incremental enhancement of the on current resulted from a slight oxygen doping when the F-WSe_2_ was exposed to air, which resulted in an incremental enhancement of mobility as a function of the air exposure time, which is consistent with the previously reported paper [27].

As shown in Figure 7c, the field-effect mobility increased by 6.6 cm^2^/V⋅s with increasing air exposure time. As shown in Figure 7d, the threshold voltage remained almost constant. Thus, the increase in mobility and I_ds_ during aging may be due to W oxidation-induced p-doping by oxygen atoms [41].

The performance of the doped WSe_2_ transistor did not deteriorate as the time of exposure to air increased. For a given sample, the passivation effect of Cytop was consistently observed (Figure S4). To investigate the Cytop encapsulation effect, we compared the transfer curve measurements in a vacuum (<2 × 10^−3^ torr) and air for samples annealed with the pristine WSe_2_ and F-WSe_2_ at 200 °C, respectively (Figure S5). The pristine WSe_2_ exhibited a variation in the transfer curve depending on the atmospheric environment (i.e., air and vacuum). This means that the pristine WSe_2_ device was vulnerable to air, as shown in Figure S5a [42]. In contrast, the transfer curve of F-WSe_2_ in Figure S5b maintained its characteristics unchanged by the air exposure effect, which is consistent with the previously reported studies using Cytop [27,38]. The hydrophobic surface energy of Cytop can protect the device from water and oxygen molecules, which can cause unwanted leakage effects through the 2D semiconductor surface [43,44]. The Cytop coating protects the surface of the WSe_2_ channel, and it was confirmed that the p-doping performance could be maintained for a long time by the Cytop encapsulation.

## 4. Conclusions

A simple and stable p-doping technique was proposed by coating WSe_2_ with Cytop, an amorphous fluorinated polymer. This p-doping phenomenon is characterized by a positive *V_th_* shift and on/off current ratio, as well as improved hole mobility, resulting from the effect of the Cytop C–F bond dipole on the WSe_2_ surface. At a higher annealing temperature, more hole carriers were induced, as the C–F bonds were aligned at higher *T_A_*. This in turn induced stronger hole accumulation in WSe_2_ and lowered the Fermi level of WSe_2_. The observed p-doping mechanism was explained by analysis of the variation in the AFM images and Raman spectra according to the annealing temperature. Furthermore, because the hydrophobicity of Cytop protects the surface of WSe_2_ from the surrounding environment, it was confirmed that the p-doping effect of Cytop was maintained for a long time even in air, with a small change in device performance compared to the initial doping state. The combination of p-doping and WSe_2_ with Cytop provides a new and promising solution for obtaining high-performance p-FETs with TMD semiconductors.

## Data Availability

Not applicable.

## Supplementary Materials

The following are available online at https://www.mdpi.com/article/10.3390/polym13071087/s1, Figure S1: AFM image of F-WSe_2_ device flake and its thickness profile. Figure S2: Transfer curves at *V_ds_* = −1 V of (a) the pristine WSe_2_ device (*T_A_* = 100, 200, 300 °C), (b) the F-WSe_2_ device (*T_A_* = 100, 200, 300 °C), (c) comparing transfer curves between the pristine WSe_2_ and the F-WSe_2_ at the same annealing condition *T_A_* = 200 °C. Figure S3: (a)–(j) transfer curves (*I_ds_-V_gs_*) for 10 pristine WSe_2_ and F-WSe_2_ devices used in the histogram. After measuring the transfer curves of the pristine WSe_2_ devices, the F-WSe_2_ devices coated with Cytop were annealed at *T_A_* = 100, 200, or 300 °C for 30 min to acquire the transfer curve data again. Using this investigation, the effect of Cytop doping on the electrical characteristics was investigated. Figure S4: (a) and (b) transfer curves of two F-WSe_2_ (*T_A_* = 200 °C) devices during 25 d exposure to air. This shows that in air, the electrical properties can be maintained over time. Figure S5: (a) Transfer characteristics of pristine WSe_2_ (*T_A_* = 200 °C) in air and vacuum. (b) Transfer characteristics of F-WSe_2_ (*T_A_* = 200 °C) in air and vacuum. Figure S6: Surface morphology of (a) pristine WSe_2_ film, and (b) WSe_2_ annealed at 300 °C for 30 min. The scale bar is 500 nm. Figure S7: Optical microscope images of (a) F-WSe_2_ (*T_A_* = 300 °C), (b) pristine WSe_2_ (*T_A_ =* 500 °C), (c) F-WSe_2_ (*T_A_* = 500 °C), respectively. Table S1: Comparison of electrical characteristics of high-performance WSe_2_ devices. Table S2: Raman shift comparison for TMD doping.
