# Supplementary material for: Interfacial Doping Effects in Fluoropolymer-Tungsten Diselenide Composites Providing High-Performance P-Type Transistors
Source: Polymers (Basel). 2021 Mar 30;13(7):1087. doi: 10.3390/polym13071087 (PMC8037493; doi:10.3390/polym13071087)
Supplement: Supplementary file 1 [file polymers-13-01087-s001.pdf]

polymers

*Supporting Information*

# Interfacial Doping Effects in Fluoropolymer-Tungsten Diselenide Composites Providing High-Performance P-type Transistors

*Hyeonji Lee*[1], *Seongin Hong*[*,2], *and Hocheon Yoo*[*,1]

Hyeonji Lee, Prof. Hocheon Yoo*

[1] Department of Electronic Engineering
Gachon University, 1342 Seongnam-daero, Seongnam 13120, Korea
E-mail: hyoo@gachon.ac.kr (H. Yoo)

Dr. Seongin Hong*
[2] School of Advanced Materials Science and Engineering
Sungkyunkwan University, Sunwon 440-746, Korea
E-mail: mindbrain@skku.edu (S. Hong)

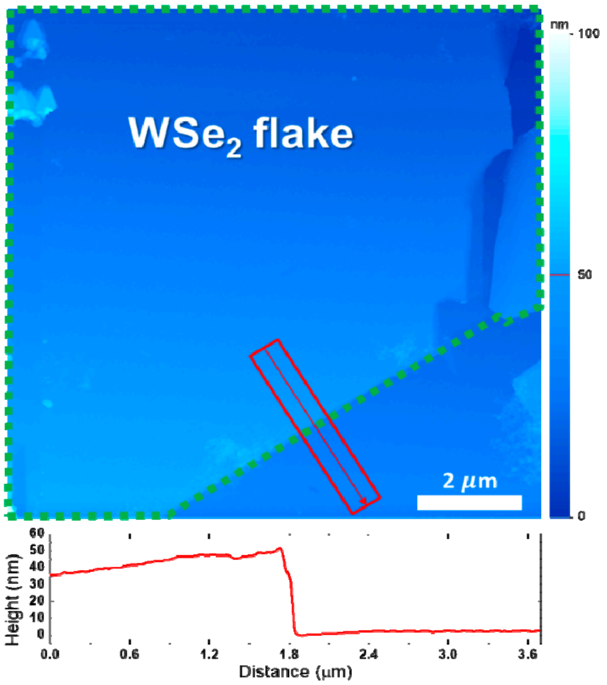


**Figure S1**. AFM image of F-$WSe_2$ device flake and its thickness profile.

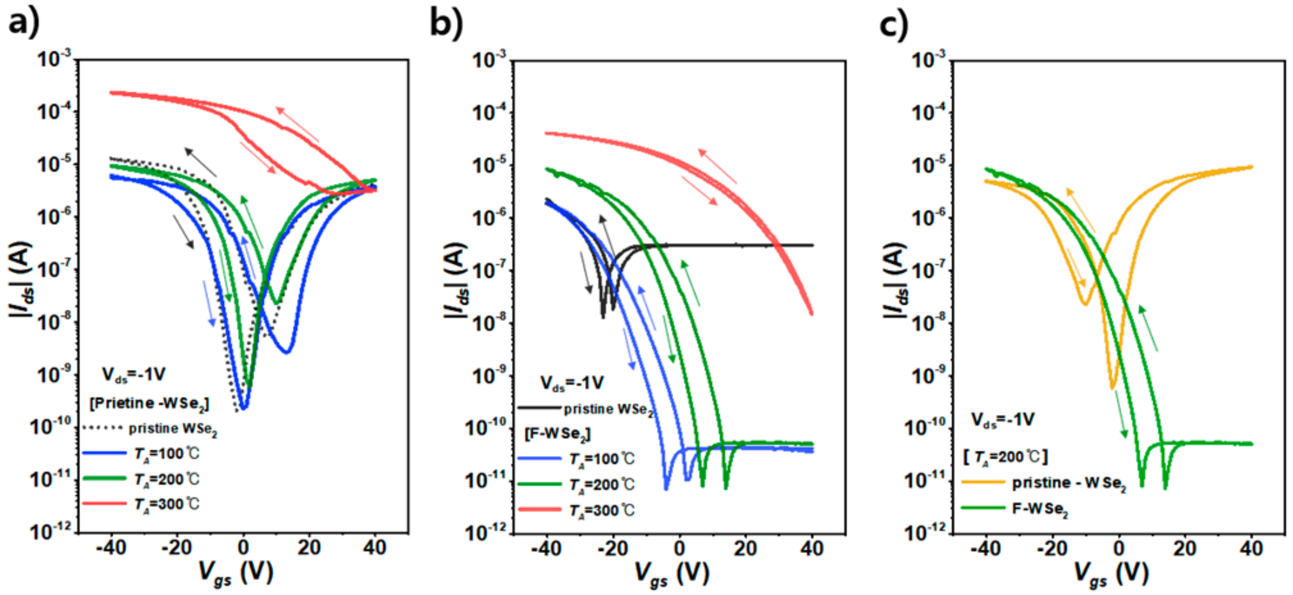


**Figure S2**. Transfer curves at $V_{ds}$ = -1 V of (a) the pristine $WSe_2$ device ($T_A$ = 100, 200, 300°C), (b) the F- $WSe_2$ device ($T_A$ = 100, 200, 300°C), (c) comparing transfer curves between the pristine $WSe_2$ and the F- $WSe_2$ at the same annealing condition $T_A$ = 200 °C

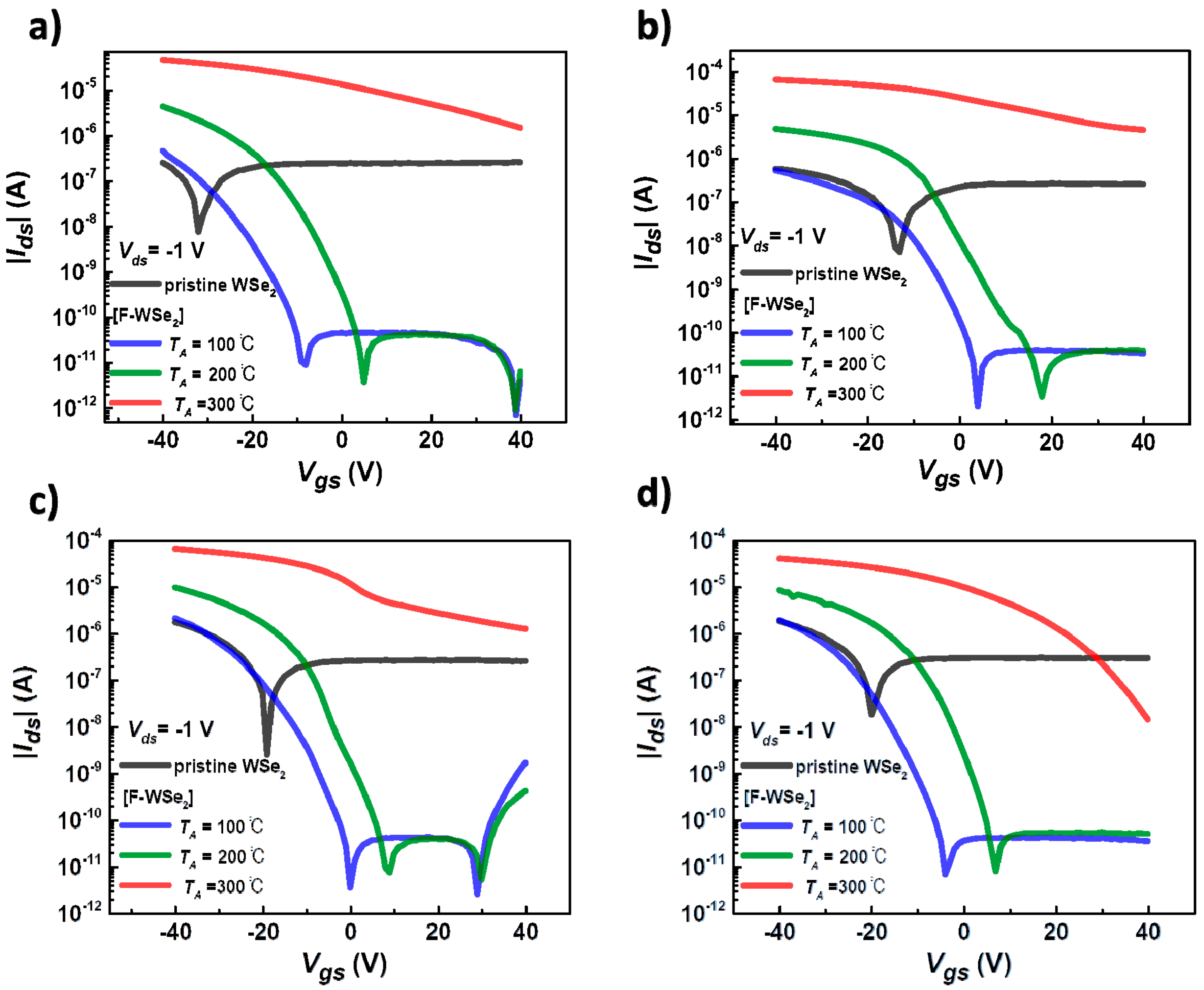

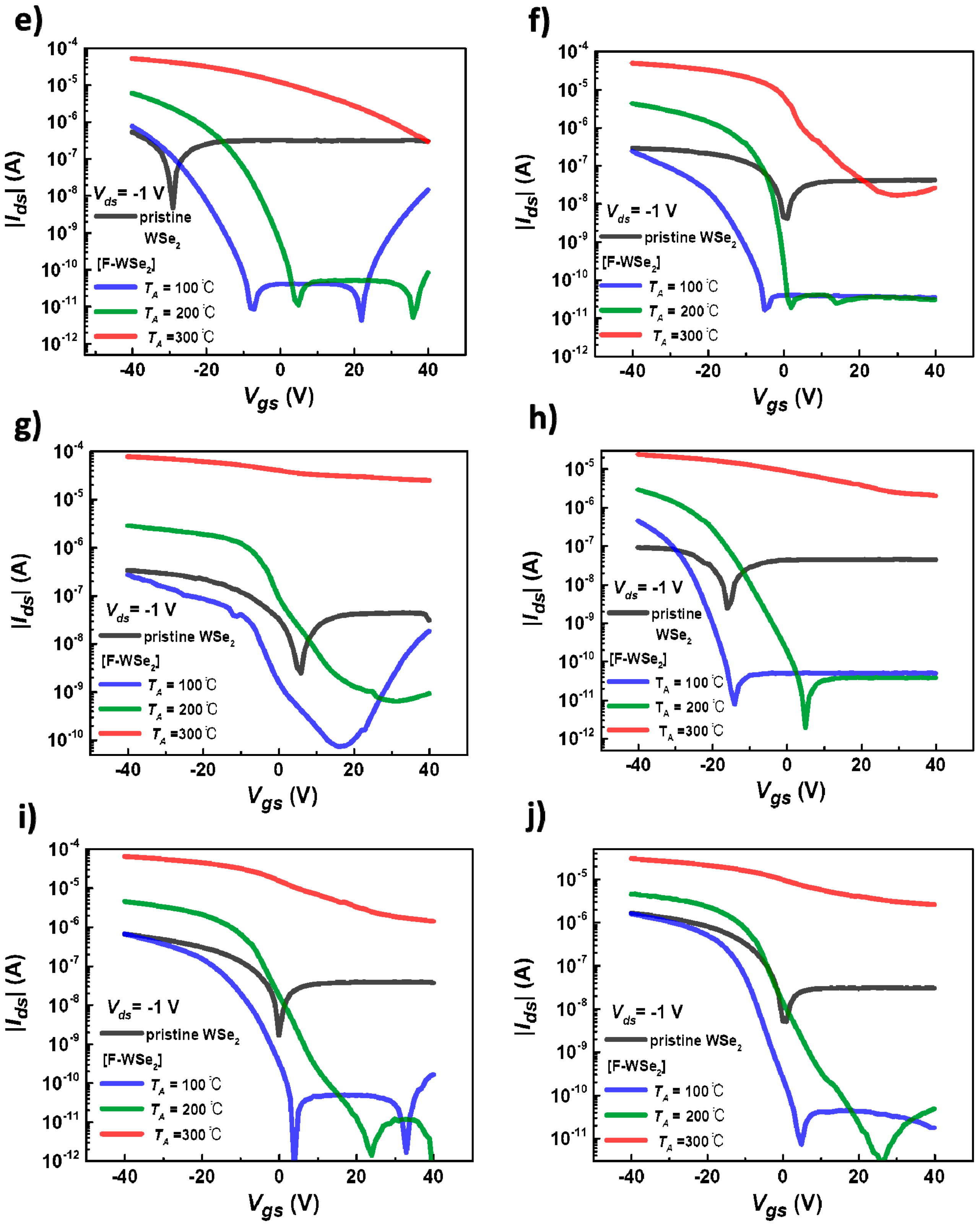


**Figure S3.** (i)-(j) are transfer curves ($I_{ds}$-$V_{gs}$) for 10 pristine $WSe_2$ and F-$WSe_2$ devices used in the histogram. After measuring the transfer curves of the pristine $WSe_2$ devices, the F-$WSe_2$ devices coated with Cytop on the devices were annealed at $T_A$ = 100, 200 or 300 °C for 30 min to measure the transfer curve again. Using this investigation, the tendency of the electrical characteristic changes on the Cytop doping effect was investigated.

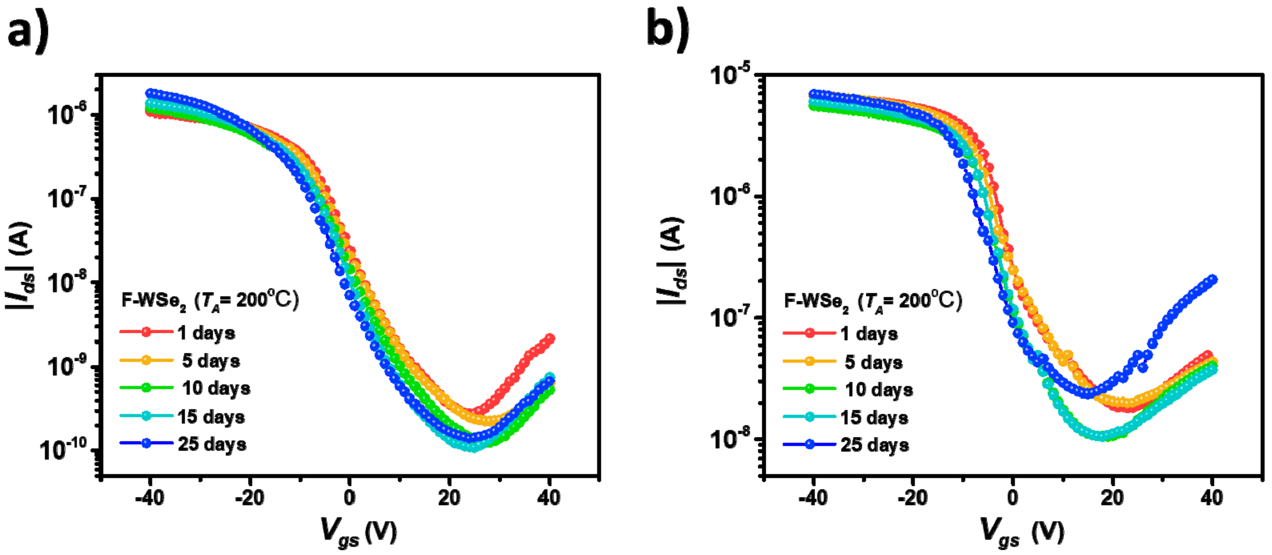


**Figure S4.** (a), (b) Transfer curves for 25 days in air in two F-$WSe_2$ ($T_A$ = 200 °C) devices. It shows that in air, the electrical properties can be maintained over time.

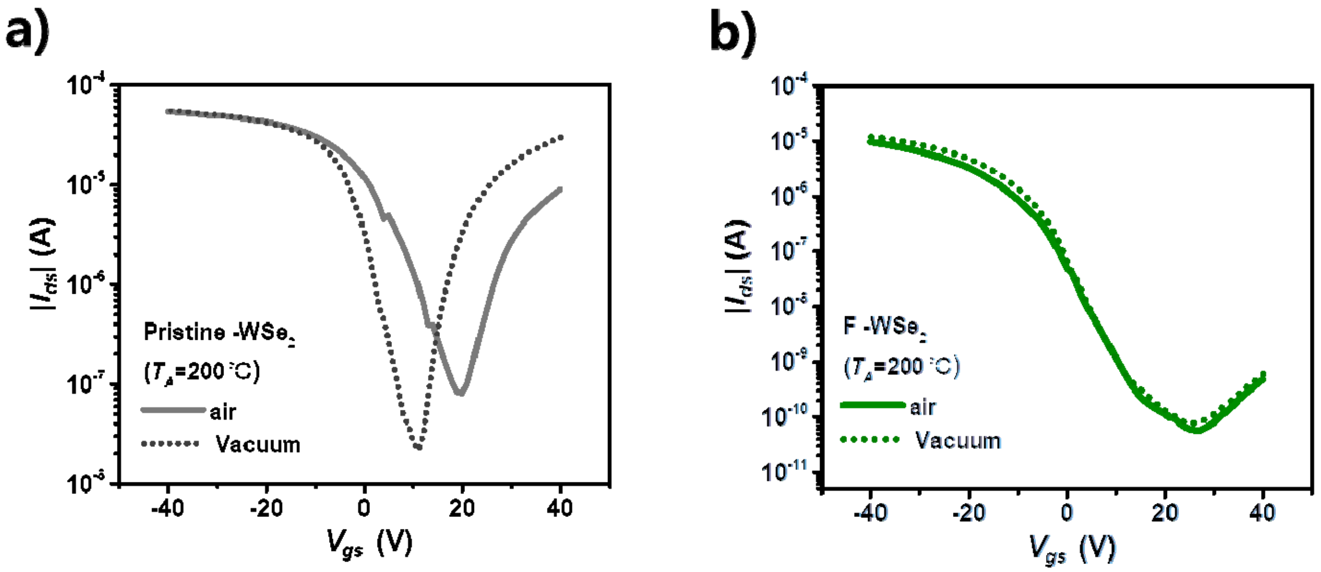


**Figure S5.** (a) Transfer characteristics of pristine $WSe_2$ ($T_A$=200°C) in air and vacuum (b) Transfer characteristics of F-$WSe_2$ ($T_A$=200°C) in air and vacuum

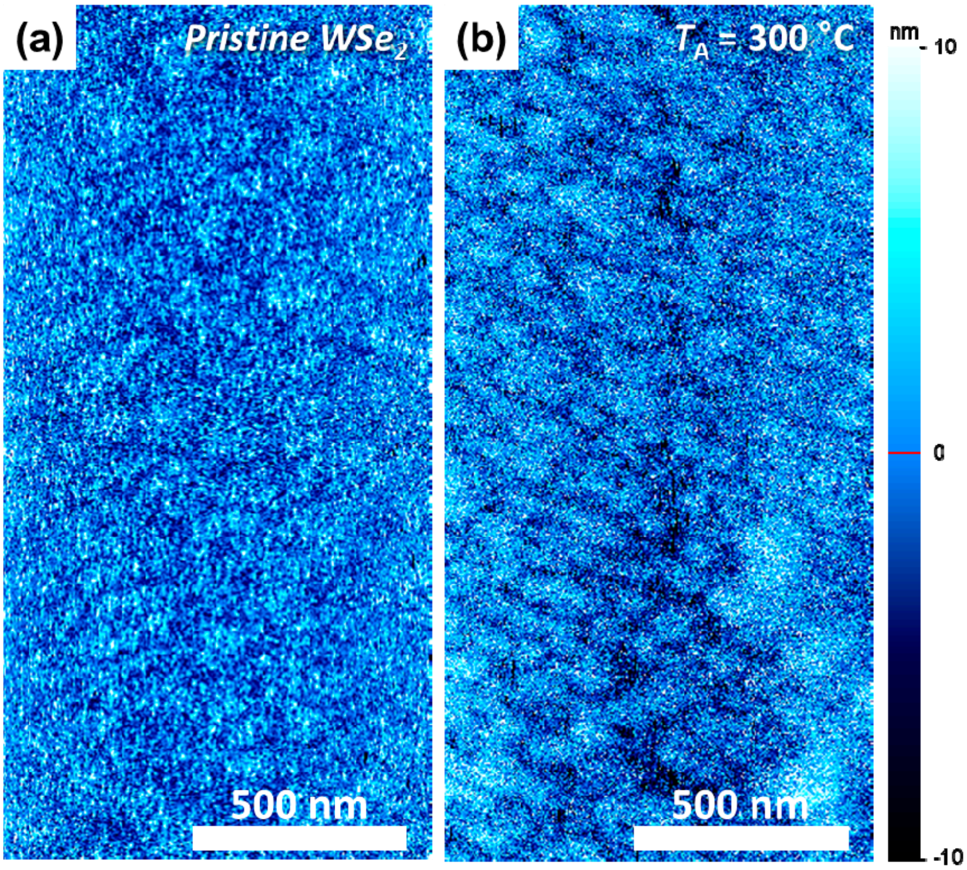


**Figure S6.** Surface morphology of (a) pristine $WSe_2$ film and (b) $WSe_2$ annealed at 300 °C for 30 min. The scale bar is 500 nm.

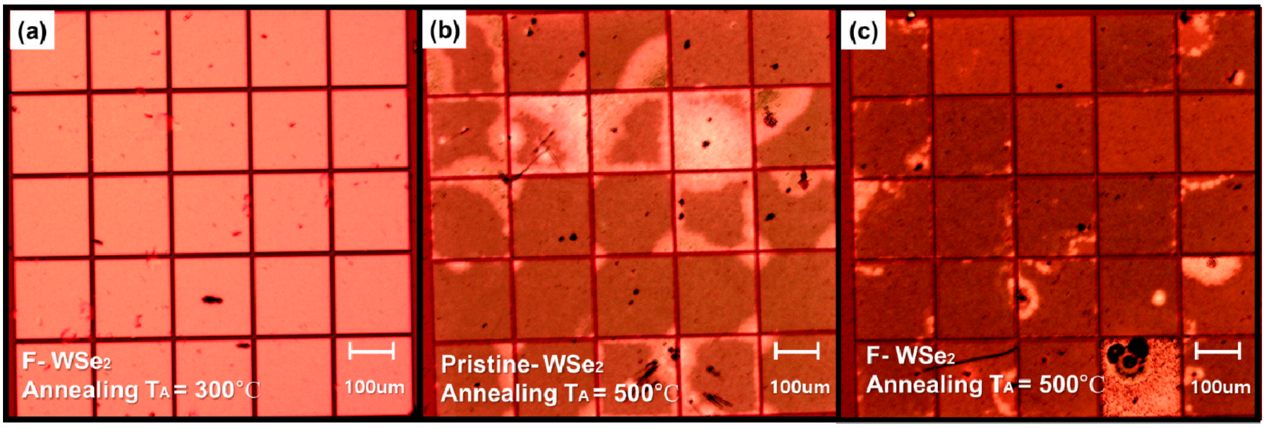


**Figure S7**. Optical microscope images of (a) F-$WSe_2$ ($T_A$=300°C), (b) pristine $WSe_2$ ($T_A$=500°C), (c) F-$WSe_2$ ($T_A$=500°C), respectively.

| Device | Contact metal | Current on/off ratio ($I_{on}/I_{off}$) | Maximum Hole mobility ($cm^2\ V^{-1}\ s^{-1}$) | Bias stress | Air stability | Hysteresis | Ref. |
|---|---|---|---|---|---|---|---|
| **F-$Wae_2$ ($T_A$=200°C)** | Ti/ Au | $10^6$ | 85 | O | 25 days | O | This Work |
| **$WSe_2$ - PFS** | Au | $10^5$ | 28 | N/A | 14 days | N/A | [S1] |
| **$WSe_2$-Ozone** | Cr/Au | $10^6$ | 41.4 | N/A | N/A | N/A | [S2] |
| **$WSe_2$- Oxygen plasma** | Cr/Au | $10^6$ | 71 | N/A | 90 days | N/A | [S3] |
| **BN- $WSe_2$** | Ti/Pb | $10^7$ | 83 | N/A | N/A | N/A | [S4] |
| **WSe2 - UV/ozone** | Ti/Pt | $10^8$ | 72.9 | N/A | N/A | N/A | [S5] |
| **WSe2- O3** | Ti/Au | $10^7$ | 31 | N/A | 24h | O | [S6] |

**Table S1** | Comparison of electrical characteristics of high-performance $WSe_2$ devices.

| TMDs | Dopant | | Raman peak shift ($cm^{-1}$) $A_g$ | Raman peak shift ($cm^{-1}$) $E_g$ | Ref. |
|---|---|---|---|---|---|
| **$WSe_2$** | Cytop | $T_A$=100°C | 0.004 | 0.003 | This work |
| | | $T_A$=200°C | 0.64 | 0.13 | |
| | | $T_A$=300°C | 1.26 | 1.25 | |
| **$ReSe_2$** | HCl | | 0.24 | 0.14 | [S7] |
| **$WSe_2$** | HCl | | 0.25 | 0.25 | [S8] |
| **$WSe_2$** | OTS | | / | 0.372 | [S9] |
| **$MOS_2$** | APTES | | 0.8 | 0.9 | [S10] |
| **$MoS_2$** | $N_2$ plasma | | 0.43 | / | [S11] |
| **$MoS_2$** | $PPh_3$ | | 0.78 | 0.78 | [S12] |
| **$WSe_2$** | $PPh_3$ | | 1.13 | 0.34 | [S13] |

**Table S2** | Raman shift comparison for TMD doping.

## References


S1. Stoeckel, M.-A.; Gobbi, M.; Leydecker, T.; Wang, Y.; Eredia, M.; Bonacchi, S.; Verucchi, R.; Timpel, M.; Nardi, M.V.; Orgiu, E. Boosting and balancing electron and hole mobility in single-and bilayer WSe2 devices via tailored molecular functionalization. *ACS nano* **2019**, *13*, 11613-11622.

S2. Zhang, M.-L.; Zou, X.-M.; Liu, X.-Q. Surface Modification for WSe2 Based Complementary Electronics. *Chinese Physics Letters* **2020**, *37*, 118501.

S3. Pudasaini, P.R.; Oyedele, A.; Zhang, C.; Stanford, M.G.; Cross, N.; Wong, A.T.; Hoffman, A.N.; Xiao, K.; Duscher, G.; Mandrus, D.G. High-performance multilayer WSe 2 field-effect transistors with carrier type control. *Nano Research* **2018**, *11*, 722-730.

S4. Liu, B.; Ma, Y.; Zhang, A.; Chen, L.; Abbas, A.N.; Liu, Y.; Shen, C.; Wan, H.; Zhou, C. High-performance WSe2 field-effect transistors via controlled formation of in-plane heterojunctions. *ACS nano* **2016**, *10*, 5153-5160.

S5. Yang, S.; Lee, G.; Kim, J. Selective p-Doping of 2D WSe2 via UV/Ozone Treatments and Its Application in Field-Effect Transistors. *ACS Applied Materials & Interfaces* **2020**.

S6. Yamamoto, M.; Nakaharai, S.; Ueno, K.; Tsukagoshi, K. Self-limiting oxides on WSe2 as controlled surface acceptors and low-resistance hole contacts. *Nano letters* **2016**, *16*, 2720-2727.

S7. Kim, J.; Heo, K.; Kang, D.H.; Shin, C.; Lee, S.; Yu, H.Y.; Park, J.H. Rhenium Diselenide (ReSe2) Near-Infrared Photodetector: Performance Enhancement by Selective p-Doping Technique. *Advanced Science* **2019**, *6*, 1901255.

S8. Nam, H.-J.; Kim, J.; Park, J.-H. Wide-Range Controllable Doping of Tungsten Diselenide (WSe2) based on Hydrochloric Acid Treatment. *The Journal of Physical Chemistry C* **2017**, *121*, 14367-14372.

S9. Kang, D.-H.; Shim, J.; Jang, S.K.; Jeon, J.; Jeon, M.H.; Yeom, G.Y.; Jung, W.-S.; Jang, Y.H.; Lee, S.; Park, J.-H. Controllable nondegenerate p-type doping of tungsten diselenide by octadecyltrichlorosilane. *ACS nano* **2015**, *9*, 1099-1107.

S10. Kang, D.H.; Kim, M.S.; Shim, J.; Jeon, J.; Park, H.Y.; Jung, W.S.; Yu, H.Y.; Pang, C.H.; Lee, S.; Park, J.H. High-performance transition metal dichalcogenide photodetectors enhanced by self-assembled monolayer doping. *Advanced Functional Materials* **2015**, *25*, 4219-4227.

S11. Azcatl, A.; Qin, X.; Prakash, A.; Zhang, C.; Cheng, L.; Wang, Q.; Lu, N.; Kim, M.J.; Kim, J.; Cho, K. Covalent nitrogen doping and compressive strain in MoS2 by remote N2 plasma exposure. *Nano letters* **2016**, *16*, 5437-5443.

S12. Heo, K.; Jo, S.-H.; Shim, J.; Kang, D.-H.; Kim, J.-H.; Park, J.-H. Stable and reversible triphenylphosphine-based n-type doping technique for molybdenum disulfide (MoS2). *ACS applied materials & interfaces* **2018**, *10*, 32765-32772.

S13. Jo, S.H.; Kang, D.H.; Shim, J.; Jeon, J.; Jeon, M.H.; Yoo, G.; Kim, J.; Lee, J.; Yeom, G.Y.; Lee, S. A High-Performance WSe2/h-BN Photodetector using a Triphenylphosphine (PPh3)-Based n-Doping Technique. *Advanced Materials* **2016**, *28*, 4824-4831.
